# Uraemic Toxins Generated in the Presence of Fullerene C_60_, Carbon-Encapsulated Magnetic Nanoparticles, and Multiwalled Carbon Nanotubes

**DOI:** 10.1155/2013/168512

**Published:** 2013-09-02

**Authors:** Magdalena Popławska, Hanna Krawczyk

**Affiliations:** Warsaw University of Technology, Noakowskiego 3, 00-664 Warsaw, Poland

## Abstract

Uraemic toxins—creatol and N-methylguanidine—are generated in conversion of creatinine in water in the presence of various forms of carbon such as fullerene C_60_, carbon-encapsulated magnetic nanoparticles, and multiwalled carbon nanotubes and oxygen. The conversion degree for creatinine was different for fullerene C_60_, CEMNPs, and MWCNTs and was 9% (3.6% creatol, 5.4% N-methylguanidine), 35% (12% creatol, 23% N-methylguanidine), and 75% (16% creatol, 59% N-methylguanidine), respectively.

## 1. Introduction

Creatinine (1) (2-amino-1-methyl-5*H*-imidazol-4-one) is a metabolite of phosphocreatine (p-creatine), a molecule used as a store for high-energy phosphate that can be utilized by tissues for the production of ATP. Creatine either comes from the diet or from the synthesis *in vivo* of amino acids such as arginine, glycine, and methionine. This occurs in the kidneys and liver, although other organ systems may be involved and specific differences may exist. Creatine and p-creatine are converted nonenzymatically to the metabolite creatinine, which diffuses into the blood and is excreted by the kidneys. *In vivo*, this conversion appears to be irreversible, and *in vitro*, it is favoured because of higher temperatures and lower pH. Under normal conditions, its formation occurs at a rate that stays between constant measurable values, which vary in different diets. Nevertheless, creatinine is a useful tool for normalizing the levels of other molecules found in urine. Creatol (5-hydroxycreatinine, 2), first identified at the beginning of the 1990s [1], is the key precursor in the synthesis of N-methylguanidine (3) uraemic toxin [2–4]. Diabetic patients with chronic renal failure accumulate the creatinine oxidative metabolites such as creatol (2) and N-methylguanidine (3) in their sera [5]. N-methylguanidine (3) level, the N-methylguanidine/creatinine molar ratio, and also creatol (2) level in the serum and urine are reported to be biomarkers for oxidative stress and can provide information concerning hydroxyl radical production in patients with chronic renal failure. Approximately one million patients throughout the world suffer from chronic renal failure [6]. Uraemic toxins may accumulate to a high level in the blood of patients. Therefore it is important that during hemoperfusion, that is, the direct contact of patients' blood with sorbent, toxins are not created. Also creating new materials as sorbents should therefore be supported by contemporaneous research into their potentially adverse impact on humans and the environment. Recent development of nanotechnology and an increase in commercial interest in its products, for example, carbon nanomaterials, lead to the production of materials that can be applied in nanomedicine. In the literature there is a lot of information on the toxicity of carbon nanomaterials [7–24], but there is a lack of information about toxicity of carbon materials and metabolites as creatinine.

**Figure 1:**
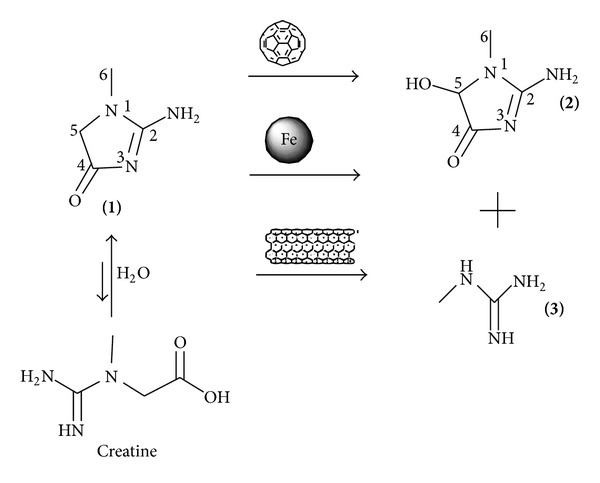
Oxidative conversion of creatinine (1) in the presence of fullerene C_60_, carbon-encapsulated magnetic nanoparticles (CEMNPs), multiwalled carbon nanotubes (MWCNTs), and oxygen into N-methylguanidine (3) via creatol (2). The rate of oxidising creatinine was faster than the rate of creatinine formation through creatinine-creatine equilibration.

Therefore, we studied the conversion of creatinine dissolved in water in the presence of charcoal and oxygen, and we obtained two toxins N-methylguanidine and creatol [25]. The goal of our studies was to check whether such a conversion occurs in the presence of other forms of carbon such as fullerene C_60_, carbon-encapsulated magnetic nanoparticles (CEMNPs), and multiwalled carbon nanotubes (MWCNTs). In Particular, the adsorption properties of creatine and vitamin B_12_ on carbon nanotubes were studied [26], but there is no information about toxins formed during this process. Studies of creatinine with fullerene C_60_, carbon-encapsulated magnetic nanoparticles (CEMNPs) and multiwalled carbon nanotubes (MWCNTs) and oxygen are described herein (Figure 1). 

## 2. Material and Methods

### 2.1. Chemistry

Fullerene C_60_ 99.9% pure was purchased from SES Research inc. Carbon-encapsulated magnetic nanoparticles (CEMNPs) with iron and iron carbide completely surrounded by protective carbon coats, containing 50% Fe and 50% carbon by weight and having diameters approx. 10–100 nm were obtained by Bystrzejewski et al. [27–30]. Multiwalled carbon nanotubes (MWCNTs) with approximately 5% Fe by weight (Thermogravimetric Analysis), diameters in the range of 15–60 nm, and lengths 1–10 *μ*m were obtained by CNT Co., Ltd. The mixture of creatinine (12.5 mg, Aldrich), fullerene C_60_ (108 mg) (or CEMNPs (50 mg) or MWCNTs (35 mg)) and freshly double-distilled water (1 mL, pH 7.0, measured by Sigma Aldrich Electrode no. Z113441-1EA) or PBS (1 mL, pH pH 7.4 ± 0.2, by Biomed, LUBLIN) was stirred at 37°C for 25 h in the presence of air. As a result, we observed the conversion of creatinine into two products (Figure 1). We could detect the presence of the new compounds just after one hour (for MWCNTs, TLC). ^1^H, ^2^H, and ^13^C NMR (nuclear magnetic resonance spectroscopy; varian VNMRS spectrometer operating at 11.7 T magnetic field, 500 MHz) spectra of the reaction mixtures were recorded, and the chemical shifts were assigned in water.

### 2.2. NMR Experiments

NMR measurements were performed on 0.55 mL samples after reaction (the mixtures were at first filtered), each one consisting of the compounds **1**, **2**, or **3** in water, to which 50 *μ*L of D_2_O containing 3-trimethylsilyl-2,2,3,3-tetradeuteropropionic acid (TSP-d_4_ sodium salt; Dr. Glaser AG Basel) was added as the spectrometer field lock and the internal chemical shift reference. The proton and proton-decoupled ^13^C NMR spectra were recorded using a Varian UNITY *Plus *spectrometer operating at 11.7 T magnetic field. The proton spectra were recorded using the following measurement parameter set: pulse angle 30°, acquisition time 5 s. 1000 scans were accumulated. This set of parameters guarantees that saturation effects are avoided. The standard measurement parameter set for ^13^C spectra was pulse width 7 *μ*s (the 90° pulse width was 12.5 *μ*s), acquisition time 1 s, spectral width 200 ppm, and WALTZ 16 ^1^H decoupling. 4000–8000 scans were accumulated, and after zero-filling to 64 K, the FID signals were subjected to Fourier transformation after applying a 1 Hz line broadening. The measurements were performed at 25°C.

### 2.3. Terms of Concentrations

 Approximately 0.2% of creatinine is oxidized by hydroxy radicals in healthy subjects to methylguanidine via creatol [31–33] (urinary levels of creatinine—1.48 mg day^−1^ and its oxidative metabolites—creatol 2.25 *μ*g day^−1^ and methylguanidine—0.39 *μ*g day^−1^). As oxidized metabolites are excreted into urine, the concentration of creatol and methylguanidine in the urine could be determined. The conversion degree for creatinine (12.5 mg) in our investigation (in tube at 37°C for 25 h) was different for fullerene C_60_, CEMNPs, and MWCNTs and was 9% Spectr. (3.6%-0.51 mg of creatol, 5.4%-0.43 mg of N-methylguanidine), 35% Spectr. (12%-1.69 mg of creatol, 23%-1.86 mg N-methylguanidine), and 75% Spectr. (16%-2.24 mg of creatol, 59%-4.77 mg of N-methylguanidine), respectively. Creatol and methylguanidine formed in much larger quantities at different carbon materials in comparison with the quantities occurring in normal nonuremic human urine (creatol: for fullerene C_60_ about 23 times more, CEMNPs about 75 times more, and MWCNTs about 100 times more; N-methylguanidine: for fullerene C_60_ about 110 times more, CEMNPs about 477 times more, and MWCNTs about 1223 times more).

## 3. Results 

It is known that in water creatinine-creatine equilibrium is achieved after one day [34]. Smith et al. [35] observed that when a creatinine solution in water was placed in contact with activated carbon in the presence of air, the concentration of creatinine in the solution continued to decrease considerably over a number of days. The authors demonstrated that at pH of dialysate and at 37°C, creatinine was rapidly oxidised. However, no creatine was produced simultaneously. In other words, the rate of oxidising creatinine on activated carbon was faster than the rate of creatinine formation through creatinine-creatine equilibration. On the other hand, Tijssen et al. [36] reported that they had observed an unknown product in the course of the contact of creatinine with activated carbon. Chemical oxidative conversion (with various active oxygen species) of creatol into N-methylguanidine is known and described in the literature [3, 4]. It has been demonstrated that creatinine in the presence of activated carbon in water is converted to N-methylguanidine *via *creatol [25]. We analysed all the published information and our investigation concerning creatinine. NMR spectra of the reaction mixtures were recorded, and the chemical shifts were assigned in water (Table 1).

**Figure 2:**
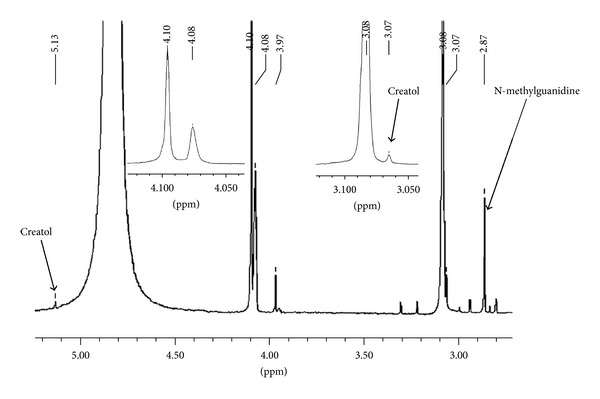
Proton spectrum of creatinine (1) after conversion with water in the presence of fullerene C_60_.

**Figure 3:**
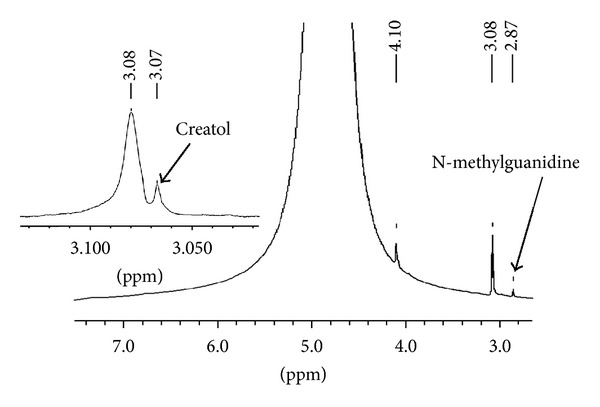
Proton spectrum of creatinine (1) after conversion with water in the presence of carbon-encapsulated magnetic nanoparticles (CEMNPs).

**Figure 4:**
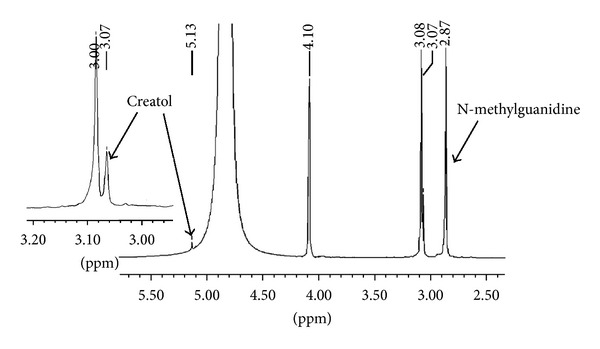
Proton spectrum of creatinine (1) after conversion with water in the presence of multiwalled carbon nanotubes (MWCNTs).

**Figure 5:**
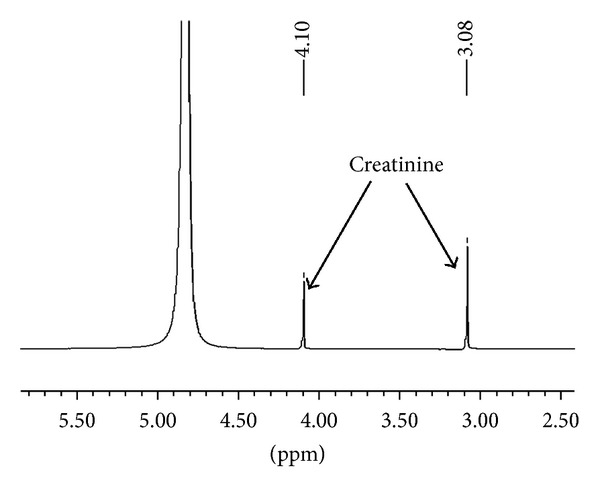
Proton spectrum of creatinine (1).

**Figure 6:**
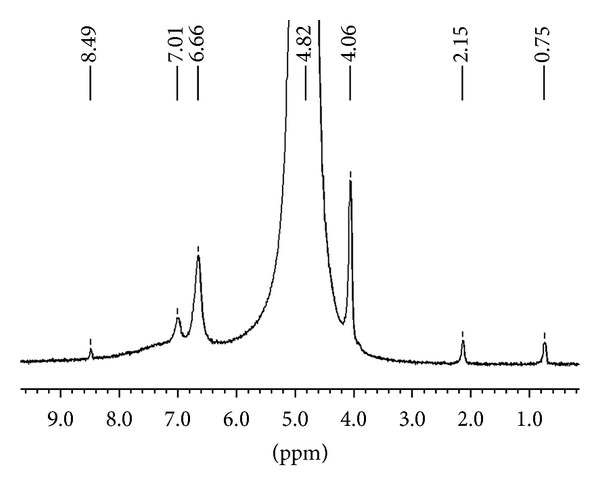
^2^H NMR spectrum of creatinine (1) after conversion with water in the presence of multiwalled carbon nanotubes (MWCNTs).

 It is known that once the intermediate (2) is formed on activated carbon, it is rapidly converted to the product (3) [13]. We observed the same phenomena in the conversion of creatinine in the presence of fullerene C_60_, CEMNPs and MWCNTs. We identified creatol (2) and N-methylguanidine (3). The conversion degree for creatinine is different for fullerene C_60_, CEMNPs, and MWCNTs and was 9% Spectr. (3.6% creatol, 5.4% N-methylguanidine), 35% Spectr. (12% creatol, 23% N-methylguanidine), and 75% Spectr. (16% creatol, 59% N-methylguanidine), respectively. In the ^1^H NMR spectra of the reactions of creatinine, water and fullerene C_60_, CEMNPs, and MWCNTs (Figures 2–4), we could observe a singlet of protons CH_3_ (*δ*, 3.08 ppm) in the unreacted creatinine (Figure 5) and, furthermore, singlets of protons CH_3_ (*δ*, 3.07 ppm) and CH (*δ*, 5.13 ppm) in creatol and a singlet of protons CH_3_ (*δ*, 2.87 ppm) in N-methylguanidine. The spectra were measured in D_2_O, and we could observe a large, broad, and residual proton signal around 4.80 ppm. In the spectra in Figures 2 and 3, singlets of protons CH_2_ (*δ*, 4.10 ppm) in unreacted creatinine (Figure 5) occurred, but in the spectrum in Figure 4 this signal disappeared. Subsequently, in the spectra in Figures 2 and 3, triplets at about *δ* 4.08 ppm with 2.5 Hz coupling constants could be observed. This signal came from the proton adjacent to the deuterium. Isotopes of an element in general are considered to have the same electronic environment. This is known as the Born-Oppenheimer approximation [37]. In these experiments, we observed ^1^ΔH(^2^H) deuterium isotope effect on ^1^H chemical shift. Creatinine has mobile protons in position 5 [38]. One of them was exchanged into deuterium, and we could observe ^1^Δisotope effect −20 ppb of CH_2_ group. For the reaction mixture with MWCNTs (in the proton spectrum this signal disappeared), we measured ^2^H NMR spectrum (Figure 6). In this spectrum, we could observe an intensive signal *δ* 4.06 ppm (4.06 + 0.02 ppm = 4.08 ppm signal in the proton spectrum). The signals *δ* 2.15 ppm and 0.75 ppm were derived from trimethylsilyl-2,2,3,3-tetradeuteropropionic acid sodium salt (TSP, internal chemical shift reference). Signals over the shift of the residual water signal came from protons connected with nitrogen. For the CH proton in position 5 of creatol, no deuterium exchange was observed (no signal was observed in ^2^H NMR spectrum). It may be noticed that in all the reactions with fullerene C_60_, CEMNPs, and MWCNTs creatine appeared (singlet, *δ* 3.97 ppm). We could assume that the reactions of creatinine with water in the presence of fullerene C_60_, CEMNPs and MWCNTs proceeded slowly and that creatine could be observed after the reactions.

In the literature, there is a lot of information on the toxicity of carbon nanomaterials [7–24]. Knowledge of the toxicity of carbon nanomaterials, particularly *in vivo*, and their impact on the environment is crucial in their potential application in nanomedicine. Preliminary tests showed that the size, surface, area, and impurities of carbon nanomaterials have a major influence on their toxicological properties. Small size and large surface area affect the chemical activity of their permeability and conductivity of biological membranes, penetration into the lungs, and absorption into the cells, which may result in the cytotoxicity of these systems [9]. It is shown that single-walled carbon nanotubes (SWCNTs) exhibit significant toxicity to human and animal cells [8], whereas multiwalled nanotubes are notably less toxic [3]. Information on the cytotoxicity of carbon nanotubes and other nanomaterials varies greatly. Sayes et al. [10] reported that under ambient conditions in water, fullerenes C_60_ could generate superoxide anions and postulated that these oxygen radicals were responsible for membrane damage and subsequent cell death. Fullerene C_60_ is toxic because it causes oxidation of lipids. It has been stated that although C_60_ has oxidative capabilities [11–17], the stability of fullerene hinders its application in medical therapy. The photosensitization of C_60_ leads to its transition into a long-lived triplet excited state. The subsequent energy or electron transfers to molecular oxygen, yielding highly reactive singlet oxygen or superoxide anion, respectively. In addition, fullerenes were found to cause chromosomal fragmentation, DNA-strand breakages, point mutations, and oxidative DNA adducts [24].

Opposite conclusions were provided by the results of Gharbi et al. [13] who showed that C_60_ is nontoxic and even protects against free radicals. Lyon et al. [17] emphasized that C_60_ is actually toxic to many bacteria, but the mechanism of toxicity is not fully known. The toxicity of various nanotubes in relation to other carbon materials has also been compared [18], demonstrating that the nanotubes are less toxic than carbon fiber and graphite and that the rise of toxicity is associated with their functionalization and formation of carbonyl or carboxyl groups on the surface of the material. Subsequent research [19] shows that the purified tubes are not poisonous, but the causes of toxicity are an amorphous carbon and a residual catalyst. Dumortier et al. [20] stated that functionalized CNT in the cycloaddition reactions were nontoxic, but the toxic tubes were oxidized or amidated, and then functionalized with polyethylene glycol. While analyzing the literature it can be concluded that it is essential to understand physicochemical properties of nanotubes and their purification methods before estimating their toxicity [21]. The contradiction of the results is mainly due to application of the tests and to different purity of carbon materials with various functional groups on the surface, as well as to conducting research on various types of cultured cells and in different conditions. An additional problem is the lack of a strict preparation procedure for the synthesis of CNTs with narrow diameter distribution, which complicates the comparison of results. Another issue is the problem of untreated nanotubes and particles containing metal crystallites [22, 23]. Nanotubes can be toxic due to metallic impurities from the process of their manufacturing. Carbon nanomaterials are a mixture containing carbon multiform and metals (Co, Fe, Ni, or Mo) occurring in different forms (pure metals, metal oxides, and metal carbides). Guo et al. [39] dealt with the problem of purifying CNTs and removal of the total metal (iron) and amorphous carbon. The authors concluded that only a small portion of the total metal in nanotubes is bioavailable, because the biological reactivity of these iron residues is possible when a portion of the metal is accessible to water chelating agents and reductants in biological media and can participate in redox reactions generating reactive oxygen species that are the molecular basis of cellular iron toxicity. It is also postulated that impurities and not CNTs themselves are responsible for the toxicity ([39, 40] and references there in).

We agree only partially with these conclusions. The reagents used in our experiments contained different amounts of iron. Fullerene contained no iron, carbon CEMNPs contained 50% iron in their interior [29, 30] (phase composition was evaluated by powder X-ray diffraction; four distinct phases were present: disordered carbon, *bcc* and *fcc*, Fe, and Fe_3_C), and MWCNTs contained 5% superficially encapsulated by carbon. In the case of CEMNPs, the conversion rate increased more than three times, in the case of MWCNTs—to over eight times more than in the case of fullerene. For pure iron (powder, diameter 5 *μ*m), no reaction was observed. It could be assumed that the iron should have been in ionic form [39] because for iron on oxidation state 0, the reaction did not occur, and the creatinine toxin was absent. It is well known that Fe(II) and Fe(III) can undergo Fenton chemistry in aqueous buffers and generate hydroxyl and superoxide radicals [3, 4]. If the iron is not in the proper oxidation state in solution, then the Fenton reaction will not occur. On the other hand, fullerenes, which do not have iron, form creatinine toxin, but in low yield. Based on our experiments, it appears that not only the impurities caused the generation of toxins, but also carbon-oxygen surface groups that are located on the surface of the carbon forms [41] affected the production of toxins. 

## 4. Conclusion

To conclude, we have shown that uraemic toxins—creatol and N-methylguanidine—are generated by conversion of creatinine in water in the presence of various forms of carbon fullerene C_60_, CEMNPs, and MWCNTs and oxygen. Preliminary tests showed that the size, surface, area, and impurities of carbon materials have a major influence on their toxicological properties. In the case of our experiments, we can conclude that the form of iron (ionic) and probably carbon-oxygen surface groups, that are located on the surface of the carbon forms, affect the production of toxins. 

Nowadays, there is a fast progress in application of carbon nanoparticles in medicine and biology. Therefore, one should take into account the possibility of generating different toxins in the presence of these materials. This information is important in the case of using different carbon forms as filters in the processes such as hemoperfusion (HP) and hemodialysis (HD).

## Figures and Tables

**Table 1:** Experimental^∗  1^H and ^13^C chemical shifts *δ* (ppm) of 5-hydroxycreatinine (**2**) and N-methylguanidine (**3**).

^ 1^H chemical shifts *δ* [ppm] of hydrogen number for compounds			^ 13^C chemical shifts *δ* [ppm] of carbon number for compounds					
**2**		**3**	**2**				**3**	
5	6	CH_3_	2	4	5	6	CH_3_	CN
5.13	3.07	2.87	171.32	190.50	85.98	30.63	30.32	160.22

*All spectra were recorded using a *Varian VNMRS* spectrometer operating at 11.7 T magnetic field (in H_2_O with TSP-d4 sodium salt in pH 7.0).
